# Application of Process Mapping to catalyze integration of HIV services in cancer care

**DOI:** 10.21203/rs.3.rs-9248594/v1

**Published:** 2026-04-03

**Authors:** Takudzwa J Mtisi, Michalina A Montano, Maganizo B Chagomerana, Agatha Bula, Wezzie Dunda, Noleb M Mugisha, Nixon Niyonzima, Cecilia Nassolo, Sibusisiwe Weza, Felix Madya, Witness Mapanga, Maureen Joffe, Ntokozo Ndlovu, Margaret Borok, Rachel Bender Ignacio, Cyrus Mugo

**Affiliations:** University of Zimbabwe; Fred Hutchinson Cancer Center; University of North Carolina at Chapel Hill; University of North Carolina Project; University of the Witwatersrand; Makerere University; Makerere University; Makerere University; University of Zimbabwe; University of Zimbabwe; University of the Witwatersrand; University of the Witwatersrand; University of Zimbabwe; University of Zimbabwe; University of Washington; Kenyatta National Hospital

**Keywords:** Cancer, HIV, Integration, Process mapping, Change, Quality improvement

## Abstract

**Introduction:**

The World Health Organization advocates for integrated health services in low- and middle-income countries, with special focus on care for non-communicable diseases within HIV care. HIV diagnosis and management with antiretroviral therapy (ART) are crucial components of whole-person care; in addition to improving overall survival, ART provides basic immunotherapy during cancer treatment. However, most cancer centers in Africa are not currently equipped to provide integrated HIV care. We conducted process mapping at referral cancer centers in Malawi, South Africa, Uganda, and Zimbabwe to identify mechanisms of integrating HIV care interventions within established cancer treatment processes.

**Methods:**

Our research consortium identified multidisciplinary clinical and non-clinical staff important to care delivery in four referral cancer hospitals and proximal HIV clinics. Consortium-level researchers met separately in-person or virtually with each site team to conduct training. High-level and detailed levels process maps were developed in process mapping sessions with diverse staff at each site and flow diagrams created to identify sites of opportunity for care integration within cancer centers.

**Results:**

Forty-five diverse clinical and non-clinical staff participated in the process across the 4 cancer centers. Several points were identified in which care integration was already occurring, including requesting HIV diagnostic and monitoring laboratory tests and clinical consideration of HIV in care plan development. While HIV care was already available in a clinic within the same hospital campus in Soweto and referral and results pathways were more integrated than the other 3 sites, none of the centers provided ART, and all lacked the capacity to provide HIV-specific counseling and management of ART or non-cancer HIV complications.

**Discussion:**

We identified multiple points at which persons in HIV care were referred for cancer treatment and where people in cancer treatment not previously known to be living with HIV or not currently on ART could be referred for HIV treatment. Some services, while not integrated, had appropriate pathways in place; for others, we identified intervention points to improve care integration of HIV services into the cancer centers. While each cancer center has distinct features, we identified general service points that could be interrogated for HIV care integration opportunities.

## Introduction

Cancer is an increasingly major public health concern in East and Southern Africa (ESA), contributing to the top three non-communicable disease causes of premature deaths among adults. Over 800,000 new cancer cases and over 500,000 cancer deaths are estimated to occur in SSA yearly, with the leading diagnoses being breast and cervical cancer for women, and prostate cancer and gastrointestinal malignancies among men(1). People with HIV (PWH) across the continent are aging and developing an increasing burden of non-communicable diseases (NCDs). This, in part, is due to widespread access to HIV treatment and prevention since 2010, which has led to the >50% reduction in new HIV acquisitions and HIV-associated deaths (2). In the four countries in which we work (Malawi, South Africa, Uganda and Zimbabwe), the HIV prevalence among cancer patients with known status in 2018–2020 is 2–4-fold that of the general population, despite most cancers being non-AIDS defining and the cancer population being older on average. In Malawi approximately 32% of cancer patients are living with HIV (vs 7% general population prevalence concurrently), 24% among mainly breast cancer patients we reviewed in South Africa (vs 13.9% l population prevalence), 29% in Uganda (vs 7.4% population prevalence), and 37% of cancer patients in Zimbabwe were living with HIV (vs 11.6% population prevalence) (3,4).

The World Health Organization advocates for integrated health services in low- and middle-income countries to realize universal health coverage, with an increasing focus on NCDs. Integration, coupled with a focus on people-centered care, envisions provision of health services as a continuum of holistic care in disease prevention, diagnosis and care at any point the patient interacts with community or facility based health system across the life course (5). To date, most integration focuses on adding NCD care and screening into HIV primary care. For cancer, this has meant focus on implementing cancer screening and triage (cervical, breast, Kaposi sarcoma) in HIV clinics, rather than the integration of HIV care into cancer specialty centers. Integration of HIV care in other services has been proposed and in some instances tested to leverage their resources to advance HIV prevention, testing and treatment goals (6). However for cancer, as is the case for tuberculosis and many opportunistic infections, antiretroviral therapy (ART) has direct implications on resolution of immune exhaustion and overall immunosuppression, which may not only decrease risk of infections, but also may play a significant role in activating endogenous anti-tumor responses (7). In addition to overt immunosuppression, HIV induces expression of immune checkpoint molecules (e.g., PD-1, CTLA-4, TIM-3), which can be partially reversed through ART (8,9). In an era in which many cancers in higher income settings, are treated with immunotherapy, and specifically immune-checkpoint inhibitors, virologic suppression for all PWH and cancer is the most basic form of immunotherapy. To advance the integration vision, there is a need for partnerships across health services to clearly understand how HIV and cancer services are offered, patient access, entry, and flow across services, and the skills of providers in the systems. Understanding these processes is crucial in the determination of optimal integration of different services. In this study we conducted process and flow mapping of select cancer care and HIV centers in four countries in East and Southern Africa to identify how HIV care services can be optimally integrated into cancer care.

## Methods

The process mapping was nested in a research study that aimed to evaluate the burden of HIV among cancer patients at cancer treatment facilities in Eastern and Southern Africa, and elucidate barriers to integration of HIV and cancer service provision as part of efforts by a new research consortium across Malawi, South Africa, Uganda, and Zimbabwe, in partnership with colleagues in Kenya and the US.

### Study sites and populations

This study was carried out in the main referral cancer centers and nearby HIV clinics in the four countries: Malawi (MW: The Malawi Cancer Centre and the Lighthouse Trust HIV Clinic), South Africa (RSA: Soweto Comprehensive Cancer Centre, Chris Hani Baragwanath Academic Hospital (CHBAH)) which also contains separate HIV clinics), Uganda (UG: Uganda Cancer Institute and The AIDS Support Organisation (TASO) Mulago), and Zimbabwe (ZW: Parirenyatwa Radiotherapy Centre and Parirenyatwa HIV Family Care Centre). The study engaged managers and healthcare providers at the cancer and HIV care clinics as contributors to the process mapping activities. Since cancer and HIV services in South Africa are both offered within an adjoining facility (and cancer clinicians can directly order HIV laboratory tests for example), the research team addressed intersections in the HIV care pathway within the cancer clinic but did not believe that separate mapping of the HIV clinic would be required.

### Human Ethics and Consent to Participate

All procedures performed in this study involving human participants were in accordance with the ethical standards of the institutional and/or national research committees, and with the 1964 Helsinki declaration and its later amendments or comparable ethical standards. The study received approval by the *University of Washington institutional review board*, and in the four countries by local ethics bodies, Malawi: *National Health Science Regional Committee (NHSRC)* and *University of North Carolina at Chapel Hill IRB*; South Africa: *University of Witwatersrand Human Research Ethics Committee*; Uganda: the *Uganda Cancer Institute Research and Ethics Committee and Uganda National Council for Science and Technology*; Zimbabwe: *Joint Research Ethics Committee (JREC) for the University of Zimbabwe Faculty of Medicine and Heath Sciences & Parirenyatwa Group of Hospitals* as well as the *Medical Research Council of Zimbabwe (MRCZ)*. All participants provided written informed consent to participate in the study.

### Study procedures

#### Training on process mapping

Research teams from the four counties were trained by author CM, an implementation scientist with experience in conducting process mapping in Kenya. Training was virtual and included: justification of process mapping in the quality improvement processes, engagement of clinic management and providers required to develop high level and detailed process maps. The training also covered guidance on the information to gather and which observations to make in the service points. The teams were also trained on the use of computer programs (Lucidchart & Microsoft PowerPoint) (10,11) for map drawing, standardized symbol designations (11) to develop site-specific maps, and the process of validating completed maps with the service providers.

#### High-level process maps

Research teams in each country held in-person or virtual 1–2 hour meetings one each with the cancer clinic management, and another with the HIV clinic management to identify the main services offered, cadres that offer the services, and understand a high-level overview of the cancer care pathway for new and returning patients. Examples of services included administrative services, clinical services, counseling services, laboratory services. Examples of cadres offering services are administrators, clinician assistants, doctors, nurses, laboratory technologists. The research teams used this information to develop high-level process maps designated as levels 1 and 2 maps with the latter providing more detail than the former.

#### Detailed process maps

These were designated as levels 3 and 4 maps. For these, the research teams utilized various combinations of frontline providers (as available) to collect detailed information in the cancer and HIV clinics regarding the respective care pathways, the services offered at specific service points and the personnel who offer the services.

In Malawi (MBC, AB & WD), the heads of the cancer and HIV clinics identified the providers that would participate in the meetings with the research team from different sub-units. There were 4 meetings in the cancer clinic and 3 meetings in the HIV clinics. During the meetings, each provider was requested to individually document in a table (standardized for all sites) the cancer or HIV care pathway, indicate services offered at different service points and the providers. Discussions were then held to arrive at a consensus *(Additional files 4–7)*, subsequently used in drawing the process maps of the care pathways.

In South Africa (WM, MJ), the head of the cancer clinic nominated a committee of providers within the clinic to provide the detailed information regarding the cancer care pathway, services offered and service provision, and crucial decision points in care. The research team then visited the different sub-units within the clinic and held brief meetings with all available providers who provided additional information or validated the information provided by the committee. The research team then cleaned the data collection tables, developed the detailed level 3 and 4 maps which they presented to the providers in the clinics in a meeting for validation.

In Uganda, the research team (NMM, NiN, CN) held in-person meetings with individual service providers at their workstations to discuss the services offered at that point, the role of different personnel at that point, the services offered or activity that preceded that service point, and the service or activity after the service point. They also provided information on crucial care decisions made at that service point, for example, if a viral load test is required, the clinician would request the patient to visit the laboratory. One in-person group meeting was held with staff representatives of every service point from each clinic to validate the information which had been given for level 1 and 2 maps as well as to arrive at a consensus on the care pathway providing information for the level 3 and 4 maps.

The research team in Zimbabwe (MZB, NN, TJM, SW, FM), held in-depth discussions with the heads of the cancer and HIV clinics. These provided an overview of the services provided in their respective clinics information which was then used to draft the level 1 process maps for the two clinics. The heads also nominated the middle level and front-line staff who were engaged in the subsequent in-depth interviews and group discussions selected on the basis of their experience and knowledge of their respective workstations. Interviews were then conducted with these individual providers at their workspaces. The insights they provided regarding the activities and processes followed by patients when accessing services in the two clinics were used to develop the level 2 process maps. Finally, two in-person group meetings were held with staff from each clinic to validate the information which had been given for level 1 and 2 maps as well as to arrive at a consensus on the care pathway providing information for the level 3 and 4 maps.

## Results

**Figure 1:**
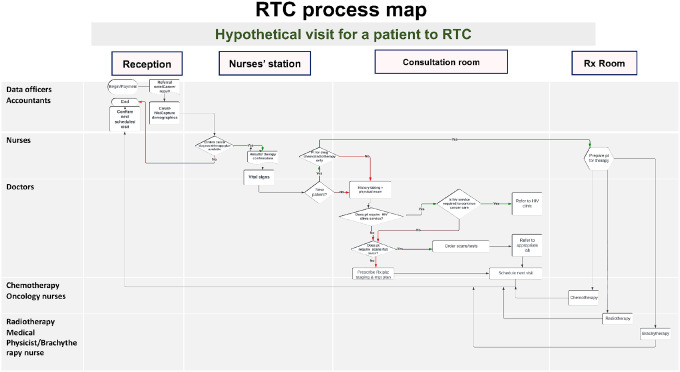
Zimbabwe Radiotherapy Centre process map and Additional files 1–3: Country process maps

Most providers involved in the process mapping in the cancer clinics were medical doctors (16/45 [36%]) and nurses (13/45 [29%]) (Table 1. *Staff involved in Process Mapping*). In general, the services provided in cancer clinics included registration by administrative clinic staff and health information officers, triage by nurses, clinical assessment by medical doctors, chemotherapy prescription by oncologists, radiotherapy by radiotherapists, laboratory services, imaging by radiologists, medication dispensing by pharmacists or pharmacy technicians and chemotherapy administration by oncology nurses (Figure 1. *Zimbabwe Radiotherapy Centre process map and Additional files 1–3: Country process maps*).

The mapping (level 4 maps) revealed several links between the cancer and HIV clinics. In Malawi, Uganda and Zimbabwe, there were clear pathways for referral of PWH suspected to have cancer from the HIV clinics to the cancer centers for diagnosis and treatment. These three countries also had established pathways for the referral of patients newly diagnosed with HIV while undergoing cancer care to the HIV clinics to initiate HIV care. The HIV clinics and cancer centers in these three countries are physically separate entities with the HIV clinics managed by a public trust (MW), a non-governmental organization (UG), and a University (ZW) respectively; cancer clinics were managed by the Ministry of Health in Malawi and Uganda, and the University of Zimbabwe in Zimbabwe. In South Africa, the Soweto Comprehensive Cancer Centre at Chris Hani Baragwanath Academic Hospital (CHBAH), an academic hospital, provides medical oncology (chemotherapy) treatment. While it is not directly connected to the HIV clinic, patients that are newly diagnosed with HIV while receiving cancer care are referred to CHBAH HIV clinic within the same hospital campus for management. Once stable on treatment they are down-referred to primary healthcare facilities within the CHBAH referral network for 3 monthly provisions of ARTs provided at no charge to patients and for 6 monthly viral load monitoring.

The following were the documented HIV services offered in the cancer care pathways [Additional files 4–7 Services and activities for cancer care delivery]. In Malawi’s cancer care, the clinical aide or nurse recorded the HIV status from a health passport or as reported by a patient during patient registration at the reception and during the clinician consultation. The clinician also recorded previous CD4+ count or viral load results from the patient’s health passport or requested the laboratory tests if they were due or not recorded. For patients of unknown HIV status, the clinician could also make a request for an HIV test for patients if clinically relevant. In Uganda, during the clinical assessment, CD4+ counts and viral load results were recorded if previously done or else a laboratory request was made for the tests. The cancer clinic also had a counseling office where a counselor provided pre-testing and post-testing HIV counseling. The laboratory in the cancer center was equipped to collect blood samples that are then transported to the nearby HIV testing center for CD4 count and viral load testing. For HIV treatment however, patients who were newly diagnosed were given referral letters to the HIV care center to start HIV care. In Zimbabwe, the service point in the cancer care pathway where HIV related services are offered was the clinician recording the CD4 and viral load results if available or making laboratory requests for the same if required for cancer treatment planning. In South Africa, HIV status, viral loads and CD4 counts were recorded during the initial oncologist consultation, and if not available, the laboratory tests were requested. Routine/scheduled viral load monitoring was also done by the clinician. In RSA, laboratory results are electronically accessible from a central repository, which meant that the clinicians in the HIV clinic could access the results of tests requested by the cancer care clinician. [Additional files 4–7 Services and activities for cancer care delivery].

The study overall found that several core HIV-related services were not commonly integrated into cancer care settings across the four countries. In particular, services essential for the ongoing management of antiretroviral therapy (ART) including onsite pharmacy services for ART refills, regimen adjustments, and structured adherence counseling were infrequently available within cancer centers. Additionally, HIV-specific counseling, which is crucial for providing cancer patients with tailored information and emotional support after an HIV diagnosis and during care, was rarely available in the cancer centers, except in UG. Where available, counseling services were largely in the HIV clinics. and did not adequately address the compounded practical challenges associated with managing concurrent HIV and cancer diagnoses. Similarly, support groups that could offer peer support and shared experiences for individuals navigating both HIV and cancer diagnoses were not widely implemented. Furthermore, formal referral pathways to peer support or patient navigation services tailored to this dual burden were largely absent.

## Discussion

The integration of HIV services in cancer care was already happening at different levels in the 4 countries. Among the integrated services, clinical assessments were the most common, with clinicians in the cancer centres requesting HIV tests as necessary, as well as CD4 and viral load tests during the cancer care planning process. Although the laboratories within some cancer centers were responsible for collecting samples for HIV-related tests, the actual processing and testing were conducted in central laboratories specifically designated for HIV-related diagnostics. Availability of HIV testing for cancer patients was largely limited to laboratory facilities in the HIV clinic and hospital central laboratory, indicating a more centralized approach to laboratory services. Notably, the accessibility of HIV test results varied between countries; in South Africa, these results were available across all HIV and cancer service points, facilitating comprehensive care, whereas in Zimbabwe, this is not the case; there is no HIV testing point in the cancer centre. When oncology teams refer patients for HIV testing at the RTC, test results are not integrated into the hospital information system, requiring patients to physically transport results back to the oncology clinic introducing delays and potential discontinuities in care. This variability in service integration highlights the differing levels of infrastructural support and resource allocation for HIV-related care within oncology settings in the region.

The lack of comprehensive HIV services within cancer care centers that was noted may limit holistic management of patients with dual diagnoses, highlighting a significant gap in integrated care that could adversely affect patient outcomes. People living with HIV who develop cancer often face substantial logistical and physical barriers to accessing parallel HIV services, including long travel distances between oncology and HIV clinics, increased financial costs, and fragmented appointment schedules. These challenges are further compounded by the effects of advanced malignancy and cancer treatment, as patients may be too unwell to travel for routine ART refills or additional clinical reviews. Moreover, cancer diagnosis and treatment may necessitate ART regimen review or modification due to potential drug-drug interactions with chemotherapy, altered organ function, or treatment-related toxicities.

When ART services are not embedded within cancer care settings, opportunities for timely regimen optimization may be missed, increasing the risk of adverse events, virologic failure, or treatment interruption. Similarly, the absence of integrated adherence counseling within oncology services overlooks a critical period of vulnerability, as cancer-related symptoms, psychological distress, and treatment side effects can significantly disrupt ART adherence. Collectively, these gaps underscore how siloed HIV and cancer care models may inadvertently exacerbate morbidity among people living with both conditions, highlighting the need for patient-centred, integrated service delivery models that align HIV treatment management with oncology care, particularly for those with advanced disease or limited access to decentralized services. Addressing these service gaps could enhance the continuum of care for patients, ensuring that all cancer patients receive a timely HIV diagnosis if their status is unknown, and that all PWH receive uninterrupted ART and treatment monitoring alongside their cancer treatment.

Peer support interventions, including structured patient counseling and navigation, may therefore present a promising strategy to support individuals living with both HIV and cancer in navigating complex care pathways. In HIV care settings, integrated adherence counseling, peer support, and patient navigation models have been shown to improve engagement in care, treatment adherence, and patient-centered outcomes, suggesting their potential relevance for integrated HIV–cancer care delivery(12–14).

The process mapping exercise provided moments of reflection by service providers, with the four country teams acknowledging opportunities to catalyze changes in the cancer care pathways to optimize cancer and HIV service delivery. In Zimbabwe for example, the providers indicated that a fully optimized integration of HIV services in cancer care units was possible, but it would require significant planning and funding as well as capacity building of staff. As a starting point it would be necessary to create linkages with clear follow up procedures for referral between the two clinics for patients to easily access and navigate the services. The frontline providers envisioned a full-scale integration with all HIV services offered to patients undergoing cancer care under one roof, in the spirit of comprehensive cancer care. To do this requires resource mobilization and planning to ensure effective implementation.

Moving forward, there is a need to clearly identify barriers to integration of the HIV services in the different steps of the cancer care pathway, and implementation strategies to overcome these barriers(15). Subsequently, evaluation of the most feasible and impactful strategies should be performed to support the development of implementation roadmaps aimed at scale. In this process, there will be questions relating to funding for HIV services, which in most sub-Saharan African countries are funded as vertical programs by partners to Ministries of Health (e.g., PEPFAR and Global Fund) and whether some of these funds can be moved to cancer care centers to improve capacity building of providers to test and appropriately treat HIV, and stocking of cancer pharmacies with antiretroviral therapy medications(16). However, this comes in the wake of recent reductions in HIV funding thereby increased resource scarcity affecting countries with the highest global HIV prevalence(17).

### Strengths and Limitations

A key strength of this study is the multi-country, multi-site collaborative approach, which enabled evaluation of HIV- cancer care integration across diverse health system contexts. Although participating sites were among the larger and relatively better-resourced cancer centers within their respective countries, they represent important referral hubs where integration efforts are most likely to be initiated and scaled. To our knowledge, this work represents one of the first systematic attempts to apply structured process-mapping and implementation-focused methodologies to assess and support integration of HIV and cancer care in low- and middle-income country settings. By leveraging cross-site collaboration and shared learning, the study advances an implementation-oriented framework for identifying service delivery gaps and opportunities for integrated care, providing a foundation for future intervention development in this emerging area.

This study also has several limitations that warrant consideration. The inclusion of heterogeneous sites across multiple countries enhances the breadth of implementation experience captured; however, this diversity limits the ability to generate a single, standardized set of recommendations applicable across all participating settings or readily transferable to other institutions or countries. Furthermore, because participating centers were relatively well-resourced compared with many cancer care facilities in the region, findings may not fully reflect constraints faced by smaller or less-resourced sites. Nevertheless, the implementation approach and methodological framework presented offer generalizable guidance on strategies for systematically identifying integration gaps and potential intervention points, even if the specific solutions require local adaptation.

## Conclusions

The process mapping identified multiple points at which persons with HIV and cancer were referred for treatment and conversely, where people in cancer treatment not previously known to be living with HIV or those needing to initiate or continue receiving ART could do so. Some services, while not integrated, had appropriate pathways in place; for others, we identified intervention points to improve care integration of HIV services into the cancer centers. Our evaluation provides insight into distinct features at each cancer center and identified general service points that could be interrogated for HIV care integration opportunities in cancer treatment facilities in other LMICs in which cancer and HIV care are provided in siloed vertical systems.

## Supplementary Material

Supplementary Files

This is a list of supplementary files associated with this preprint. Click to download.

- Additionalfile1.SouthAfricaprocessmap.pdf
- Additionalfile2.Ugandaprocessmap.pdf
- Additionalfile3.MalawiCancerprocessmap.pdf
- Additionalfile4.ServicesandactivitiesforcancercaredeliveryZimbabwe.docx
- Additionalfile5.ServicesandactivitiesforcancercaredeliverySouthAfrica.docx
- Additionalfile6.ServicesandactivitiesforcancercaredeliveryUganda.docx
- Additionalfile7.ServicesandactivitiesforcancercaredeliveryMalawi.docx

## Figures and Tables

**Table 1:** Staff Involved in Process Mapping at Regional Cancer Referral Centers and Proximal HIV Clinics in Malawi, South Africa, Uganda, and Zimbabwe

	Cancer Ci	inters			HIV clinics			
Roles	Malawi	South Africa	Uganda	Zimbabwe	Malawi	South Africa	Zimbabwe	Uganda
**Clinicians**	7 doctors 3 clinical officers	1 doctor	5 medical officers 1 clinical officer 2 oncologists 1 radiation oncologist	2 oncologists 2 oncology residents	6 clinic officers	NA	2 doctors	2 doctors
**Nurses**	8 nurses	3	2 cancer screening 1 navigation	2	6	NA	1 charge nurse 1 clerking nurse	4
**Pharmacists**	2	1 pharmacist 1 assistant	2	NA	NA	NA	1	1
**Data or Administrative Clerks**	3	NA	1	2	NA	NA	1	1
**Counselors**	NA	NA	1	NA	NA	NA	1	NA
**Laboratory Staff**	NA	NA	4	NA	NA	NA	NA	NA
**Other Staff**	NA	NA	NA	1 radiographer	5 clinic aides	NA	NA	NA

Process mapping of integration of HIV care into cancer care involved separate mapping of each of the cancer referral centers (The Malawi Cancer Centre inn Lilongwe, Malawi; Soweto Comprehensive Cancer Centre in Soweto, Gauteng, South Africa; Uganda Cancer Institute in Kampala, Uganda; and Parirenyatwa Radiotherapy Clinic in Harare, Zimbabwe), and at geographically proximal HIV clinics, either under the same institution or with whom there is a referral relationship (Lighthouse Trust HIV clinic in Lilongwe; The AIDS Service Organization (TASO) Mulago in Kampala; and Parirenyatwa HIV Family Care Centre in Harare; there was no separate process mapping done for the HIV at Chris Hani Baragwanath Academic Hospital, which is within the same medical center campus as the Soweto Comprehensive Cancer Centre). Process mapping was facilitated by research staff with the inclusion of the above-delineated staff roles represented from each participating institution.
